# The Medical Association of the State of Alabama

**Published:** 1890-05

**Authors:** 


### THE MEDICAL ASSOCIATION OF THE STATE OF ALABAMA.

The annual Session of the Medical Association of the State of Alabama, for the current year, was held in the city of Birmingham, commencing April (8th), at 12 o'clock, noon, and continuing four days.

Regular reports were submitted on the following subjects, viz:

(1.) Benjamin James Baldwin, M. D., Montgomery—Headache and Neuralgia, Resulting from Refractive Errors.

(2.) Enoch James Conyngton, M. D., Decatur—Perineal Lacerations and their Treatment.

(3.) William Elias Brownlee Davis, M. D., Birmingham—A study of the Treatment of Local and General Peritonitis.

(4.) John Brown Gaston,, M. D., Montgomery—Cerebro-spinal Fever.

(5.) Samuel Maris Hogan, M. D., Union Springs—Congenital Club-foot and its Early treatment.

(6.) Bolivar Thomas Jones, M. D., Huntsville—Antisepsis in Surgery.

(7.) Cristo Americus Robinson, M. D., Huntsville—Antisepsis in Surgery.

(8.) Edward Henry Sholl, M. D., Birmingham—The Practical Relation of the Physician to Life Insurance.

(9.) James Grey Thomas, M. D., Mobile—Some observations made during a Trip Abroad.

(10.) Isaac LaFayette Watkins, M. D., Montgomery—The Therapeutics of the Endometrium.

(11.) James Anthony Wilkinson, M. D., Flomaton—Some Thoughts on our Modern Therapeutics.

The third day was given up to the Omnibus Discussion, during which the following volunteer papers were read and discussed:

T. W. Ayres, M. D., Jacksonville, Ala.,—Therapeutic Value of Phenacetine—W. H. Hudson, LaFayette.

Tuberculosis as an Infectious Baccillary Disease, and its Relation to Hygiene—J. E. Burdon, M. D., Cullman.

Physical Research and Practical Medicine—H. M. Hunter, M. D., Union Springs.

Placenta Prævia—W. M. Wilkinson, M. D., Montgomery.

Exophthalmic Goitre—J. R. Jordan, M. D., Montgomery.

Some Remarks on Rheumatism in Infant Life—J. H. Blue, M. D., Montgomery.

Pathological Significance of Albumen in the Urine,—J. P. Furniss, M. D., Selma.

The Continued Fevers in Alabama—E. W. Morris, M. D., Birmingham.

Treatment of Organic Stricture of the Urethra—B. L. Wyman, M. D., Birmingham.

Treatment of Hemorrhoids with special Reference to Operative procedure.

---

CHEST PAINS.—For the troublesome pains located under the sternum, and elsewhere in the chest, frequently complained of in bronchitis, M. W. Emerson, M. D., has found this formula of much value:

℞. Sodii salicylatis,
Potassii nitratis,
Pulv. ipecac. et opii - - - aa gr. ij.
Fiat capsula - - - - - j.

Sig. Every three hours.

—*College and Clinical Record.*

